# Differential Expression Analysis of Olfactory Genes Based on a Combination of Sequencing Platforms and Behavioral Investigations in *Aphidius gifuensis*

**DOI:** 10.3389/fphys.2018.01679

**Published:** 2018-11-27

**Authors:** Jia Fan, Qian Zhang, Qingxuan Xu, Wenxin Xue, Zongli Han, Jingrui Sun, Julian Chen

**Affiliations:** The State Key Laboratory for Biology of Plant Diseases and Insect Pests, Institute of Plant Protection, Chinese Academy of Agricultural Sciences, Beijing, China

**Keywords:** *Aphidius gifuensis*, full-length transcriptome, pheromone perception, ORs, IRs, OBPs, CSPs

## Abstract

*Aphidius gifuensis* Ashmead is a dominant endoparasitoid of aphids, such as *Myzus persicae* and *Sitobion avenae*, and plays an important role in controlling aphids in various habitats, including tobacco plants and wheat in China. *A. gifuensis* has been successfully applied for the biological control of aphids, especially *M. persicae*, in green houses and fields in China. The corresponding parasites, as well as its mate-searching behaviors, are subjects of considerable interest. Previous *A. gifuensis* transcriptome studies have relied on short-read next-generation sequencing (NGS), and the vast majority of the resulting isotigs do not represent full-length cDNA. Here, we employed a combination of NGS and single-molecule real-time (SMRT) sequencing of virgin females (VFs), mated females (MFs), virgin males (VMs), and mated males (MMs) to comprehensively study the *A. gifuensis* transcriptome. Behavioral responses to the aphid alarm pheromone (E-β-farnesene, EBF) as well as to *A. gifuensis* of the opposite sex were also studied. VMs were found to be attracted by female wasps and MFs were repelled by male wasps, whereas MMs and VFs did not respond to the opposite sex. In addition, VFs, MFs, and MMs were attracted by EBF, while VMs did not respond. According to these results, we performed a personalized differential gene expression analysis of olfactory gene sets (66 odorant receptors, 25 inotropic receptors, 16 odorant-binding proteins, and 12 chemosensory proteins) in virgin and mated *A. gifuensis* of both sexes, and identified 13 candidate genes whose expression levels were highly consistent with behavioral test results, suggesting potential functions for these genes in pheromone perception.

## Introduction

*Aphidius gifuensis* Ashmead is a dominant endoparasitoid of aphids such as *Myzus persicae* and *Sitobion avenae* (Ohta and Honda, 2010) and is best known for its use in the control of tobacco aphids in China. Due to interest in the biocontrol properties of this species, the ecology and biology of *A. gifuensis* have been extensively studied. Generally, *A. gifuensis* can start mating 30 min after emergence. Females mate only once in a lifetime, whereas males mate repeatedly (Bi and Ji, 1994). During mating, female-borne cues are found to be responsible for eliciting courtship behaviors from male wasps (e.g., Bi and Ji, 1994). Olfactory cues are also critical for parasite searching behavior. For example, E-β-farnesene (EBF), a common active component of the alarm pheromone in aphids, can be tracked by *A. gifuensis* as a kairomone to locate potential target aphids (e.g., Tan and Liu, 2014).

Various odor-related proteins, such as odorant receptors (ORs), inotropic receptors (IRs), odorant-binding proteins (OBPs) and chemosensory proteins (CSPs), are responsible for specific odor selection and peripheral signal transduction in insects. OBPs and CSPs are concentrated (as high as 10 mM) in the sensillum lymph of insect antennae (Vogt and Riddiford, 1981; Pelosi et al., 2006) and are capable of carrying the semiochemical through the lymph to the ORs or IRs. Most animals, including nematodes, employ G-protein-coupled receptors (GPCR) as transmembrane ORs. The insect OR protein family was first described in *Drosophila* (Clyne et al., 1999) and was thought to comprise GPCRs as well. However, an opposite transmembrane mode compared with GPCR was later identified (Benton et al., 2006), and the insect ORs were ultimately reclassified as a novel OR protein family. Moreover, insect IRs were recently shown to play roles during insect chemical sensation in *Drosophila* (Benton et al., 2009), indicating that insect olfactory perception operates through a unique mechanism compared with that in other animals. The dual filtration by soluble proteins (OBPs and CSPs) and transmembrane receptors (ORs and IRs) therefore ensure the high sensitivity of the insect to certain odors, such as pheromones and host odors.

Previous work identified CSPs through next-generation sequencing (NGS) analysis of the antennae transcriptome (Kang et al., 2017) and represents the only molecular biological study of this species. However, little is known about the association between the behavioral responses of this wasp to chemicals and the corresponding functional genes. The molecular mechanism of chemical sensation, including olfactory perception, remains completely unknown.

The reported average lengths of the isotigs from NGS were generally <200 bp, which prevented the assembly of full-length transcripts. Single-molecule real-time (SMRT) sequencing, a third-generation sequencing platform constructed based on PacBio RS (Pacific Biosciences of California, Inc.^1^), provides long reads that are more than 4 kb in length for both genome sequencing and full-length transcriptome sequencing. Combination of SMRT sequencing with NGS reads has been shown to be ideal for accessing complete transcriptome data (Au et al., 2013).

Olfaction plays a key role in the lifecycle of *A. gifuensis*, and related ecological and physiological studies have been thorough. However, further study is needed to investigate the following hypotheses:

(1) Mated females will reject males once they mate, and olfaction plays a role in the rejection response.
(2) The male olfactory response to females before and after mating is different.
(3) The olfactory responses of females to chemical clues from aphids (such as EBF) differ before and after mating.
(4) Based on the above hypothesis, the related olfactory genes could be preliminarily uncovered through differential expression analysis of transcriptomic data of samples from both sexes before and after mating, namely, from virgin females (VFs), mated females (MFs), virgin males (VMs), and mated males (MMs).

In the present study, we combined NGS and SMRT sequencing to investigate VF, MF, VM, and MM *A. gifuensis* wasps to generate a comprehensive full-length *A. gifuensis* transcriptome. Moreover, the behavioral responses of this species to the aphid alarm pheromone and to wasps of the opposite sex were investigated in detail, enabling precise correlation of the coexpression data from the resulting transcriptional data to males (virgin or mated), which are attracted by females, and to females, which are attracted by the alarm pheromone from aphids and are parasitoids of the aphids. Accordingly, this study provides insights and is a valuable resource for further studies of olfactory mechanisms in *A. gifuensis*.

## Materials and Methods

### Insects

*Aphidius gifuensis* was originally collected from *M. persicae* mummies in August 2011 in Kunming, Yunnan province, China, and cultured in an air-conditioned insectary [25 ± 2°C60 ± 10% RH, and a photoperiod of 16:8 (L: D) h]. The mummies were collected and placed separately in petri dishes (3.5 cm in diameter). Newly emerged (within 0–12 h) male and female parasitoids were placed in petri dishes (13 cm in diameter and 2 cm in height) for 24 h (24–36-h), either separately for the virgin condition (VF or VM) or together, to allow mating, for the mated condition (MF or MM). For the mated condition, each treated *Aphidius* (MM or MF) was exposed to 10 virgin *A. gifuensis* wasps of the opposite sex to ensure that mating occurred during their stay in the petri dishes. The 24-to 36-h-old parasitoids were collected for further studies, such as transcriptome sequencing, behavioral investigation and molecular analyses. Cotton balls filled with 25% defined sugar water were constantly supplied as the diet for adult wasps.

### Behavioral Responses to EBF and Wasps of the Opposite Sex

Responses of *A. gifuensis* to EBF and wasps of the opposite sex were investigated in a *Y*-tube olfactometer. The olfactometer consisted of a *Y*-shaped glass tube with a 3-cm diameter, a 10-cm trunk length, and a 15-cm branch length. The airflow (0.1 L/min) was dried and purified using activated granular carbon and washed in distilled water before passing through a chamber where the odor source flowed into each arm (branch) of the *Y*-tube. Assays were performed as described previously (Mondor et al., 2000; Fan et al., 2015). Briefly, for each treatment, one arm was randomly selected as the treatment arm to introduce either 5 μl of freshly prepared EBF solution (400 ng/μl) or 10 wasps of the opposite sex into the odor chamber connected to the arm, while the other arm was defined as the control arm and was either used to introduce 5 μl of paraffin oil (the solvent used for EBF) or was kept empty, depending on the treatment. EBF was purchased from Wako, Japan. Mineral oil was purchased from Sigma-Aldrich, United States.

The tested insects were visually and physically separated from the odor chamber throughout testing. To prevent the wasps from escaping, bunches of fluffy and ventilated cotton were placed into both sides of the chamber as well as at the exits of both arms of the *Y*-tube olfactometer. To avoid visual disturbance, a piece of white card paper was placed between the odor chamber and the test area. The tests were conducted using 24- to 36-h postemerged *A. gifuensis* VMs, MMs, VFs and MFs in a controlled environment at 25 ± 2°C with 60 ± 10% RH, and a 16:8 (L: D) photoperiod. One *Aphidius* was released into the observed area of the olfactometer and allowed to move either until reaching one-third of the way up one of the arms or for 5 min (300 s). Two series of experiments were performed. In the first series, 10 wasps of the opposite sex of the tested *Aphidius* were loaded and allowed to move freely in the odor chamber connected to the treatment arm as the odor source. In the second series, 2000 ng (400 ng/μl, 5 μl) of EBF dissolved in mineral oil was employed (dropped onto a piece of 1^∗^1 cm^2^ filter paper) as the odor source. The EBF loaded into the treatment arm was renewed after each test, and 10 wasps of the opposite sex were kept in the odor chamber throughout the test, unless any accidental death occurred, in which case wasp replacement was necessary. Each experiment comprised 300 replications for each treatment (VF, MF, VM, MM) in each series.

### Statistical Analysis

Differences in the behavioral responses of *A. gifuensis* to the odors and blank control were determined using χ^2^ tests (SAS software 2002, SAS Institute Cary, NC, United States). Insects with no response were not included in the statistical analysis but were counted and are listed in Table 1.

**Table 1:** Response of *A. gifuensis* wasps (a) to 10 wasps of the opposite sex or a blank control and (b) to the aphid alarm pheromone EBF or a blank control.

Treatment A vs. B	Aphidius	In total	*N*(A)	*N*(B)	*N*(O)	χ2 (1 d.f.)
10 *A. geifuensis* wasps of the opposite sex (A) vs. empty control (B)	VF	300	122	129	49	0.195 NS
	MF	300	106	145	49	6.061^∗^
	VM	300	122	92	86	4.206^∗^
	MM	300	116	117	67	0.004 NS
Aphid alarm pheromone (A) vs. empty control (B)	VF	300	150	114	36	4.909^∗^
	MF	300	165	107	28	12.368^∗∗^
	VM	300	97	113	90	1.219 NS
	MM	300	149	72	79	26.828^∗∗^

*VF, virgin female; MF, mated female; VM, virgin male; MM, mated male. All Aphidius wasps emerged during the 24- to 36-h period. N(A), number of individuals who chose the treatment arm; N(B), number of individuals who chose the control arm; N(O), number of individuals who did not select either arm within 5 min. ^∗∗^P < 0.01; ^∗^P < 0.05; NS, not significant. Chi-square (χ^2^) test comparing test arm and control arm.*

### RNA Sample Preparation

Total RNA was extracted separately from *A. gifuensis* VFs, MFs, VMs, or MMs (three replicates each) using TRIzol reagent (Invitrogen, United States) according to the manufacturer’s instructions. Three-microgram RNA samples with standard quality ratios were purified using poly-T oligo-attached magnetic beads after testing the quality with an Agilent 2100 bioanalyzer.

### NGS

Divalent cations under elevated temperature in a NEB Next first-strand synthesis reaction buffer (5×) were used for fragmentation. Single-stranded (ss) cDNA was synthesized using a random hexamer primer using M-MuLV reverse transcriptase, DNA polymerase I and RNase H (NEB, United States). After adenylation of the 3′ ends of the DNA fragments, NEBNext adaptors with a hairpin loop structure were ligated to the fragments for hybridization. The library fragments were purified using the AMPure XP system (Beckman Coulter, United States) to select cDNA fragments that were 150–200 bp long. Then, 3 μl of USER enzyme (NEB, United States) were used with size-selected, adaptor-ligated cDNA at 37°C for 15 min followed by 5 min at 95°C before PCR. PCR was then performed using Phusion high-fidelity DNA polymerase, universal PCR primers and an index (X) primer. The products were purified (AMPure XP system), and library quality was assessed using the Agilent Bioanalyzer 2100 system (Agilent Technologies, United States). Clustering of the index-coded samples was performed on a cBot cluster generation system using the TruSeq PE Cluster Kit v3-cBot-HS (Illumina, China) according to the manufacturer’s instructions. The library preparations were sequenced on an Illumina HiSeq 2500 platform, and paired-end reads (the sequencing strategy was PE125) were generated after cluster generation. After sequencing, the raw reads were processed to remove low quality and adaptor sequences by NGS QC and then assembled into unigenes using Trinity r20140413p1 with min_kmer_cov:2 and the other parameters set to default values.

### SMRT Sequencing

First-strand cDNA was synthesized using the SMARTer PCR cDNA Synthesis Kit (Clontech^2^) using SMARTScribe reverse transcriptase, CDS primer IIA [5′-AAGCAGTGGTATCAACGCAGAGTACT_30_*N*_−1_*N*-3′] and SMARTer IIA oligonucleotide (5′-AAGCAGTGGTATCAACGCAGAGTACXXXXX-3′) for 14 cycles. The purified cDNA was normalized using the Trimmer-2 cDNA Normalization Kit (Evrogen^3^). Then, second-strand cDNA synthesis was performed using PrimerSTAR GXL DNA polymerase (Clontech^2^) with 5′ PCR primer IIA (5′-AAGCAGTGGTATCAACGCAGAGTAC-3′) for 18 cycles. The PCR products were purified using 0.4 × AMPure beads (Beckman^4^). Then, SMRT cell libraries were constructed using a DNA Template Prep Kit (3–10 kb, part; Pacific Biosciences of California, Inc.^1^). The templates were bound to SA-DNA polymerase and V2 primers. The complexes of the templates and polymerase were bound to magnetic beads and transferred to a 96-well PCR plate at 50 pM on-plate concentrations to reach 50% P1 for processing on a Pacific Biosciences RSII sequencing instrument using C2 sequencing reagents. The 1–2 k library was subjected to SMRT sequencing using 3 SMRT cells, the 2–3 k library was subjected to SMRT sequencing using 3 SMRT cells and the 3–6 k library was subjected to SMRT sequencing using 2 SMRT cells. Subreads were filtered and subjected to circular consensus sequencing (CCS) using the SMRT Analysis Server 2.2.0 (Pacific Biosciences of California, Inc.^1^).

### Data Processing and Annotation

The short reads generated with HiSeq 2500 were filtered using the NGS QC Toolkit. Meanwhile, the software proovread (Hackl et al., 2014) was used to correct consensus reads of the full-length transcripts by alignment with filtered NGS short reads. Redundant reads of the error-corrected consensus reads were filtered using CD-HIT-EST. Consensus reads with similarity thresholds of 0.99 were clustered, and redundant sequences were then removed. A total of 81,636 filtered nonredundant sequences were used as input data to perform the annotation. Transcriptome sequences were annotated using seven databases, namely the nonredundant protein sequence (Nr, *e*-value = 1e^−5^), non-redundant nucleotide (Nt, *e*-value = 1e^−5^), Pfam (*e*-value = 0.01), Clusters of Orthologous Groups (KOG/COG, *e*-value = 1e^−3^), Swiss-Prot (*e*-value = 1e^−5^), Kyoto Encyclopedia of Genes and Genomes (KEGG, *e*-value = 1e^−10^) and Gene Ontology (GO, *e*-value = 1e^−6^) databases.

### Quantification of Gene Expression

Gene expression levels were estimated by RSEM (Li and Dewey, 2011) for each sample: (I) Clean data were mapped back onto the transcript sequence, and (II) the read count for each gene and isoform was obtained from the mapping results.

### Differential Expression Analysis

The reads for the *Aphidius* transcriptomes from four different treatments (VF, MF, VM, and MM), with three replications for each treatment, were produced based on a combination of NGS and SMRT sequencing in this study. Expression analysis of the reads obtained from different treatments was performed using tophat and cufflinks (Trapnell et al., 2012).

Based on the results of the behavioral investigation, differential expression analyses comparing each treatment to VF and VM were separately performed using the DESeq R package (1.10.1). DESeq provide statistical routines for determining differential expression in digital gene expression data using a model based on the negative binomial distribution. The resulting *p-*values were adjusted using Benjamini and Hochberg’s approach for controlling the false discovery rate. Genes found to have an adjusted *p-*value < 0.05 by DESeq were denoted as differentially expressed genes. The log_2_(fold change) values and *p-*values are shown as a volcano plot.

Eight olfactory genes (2 ORs, 2 IRs, 2 OBPs, and 2 CSPs) were randomly selected from each family for qPCR verification of the results from the statistical analysis of the transcriptome sequencing. RT-qPCR was performed on an ABI Prism 7900 Sequence Detection System (Applied Biosystems, Warrington, United Kingdom). SYBR Green Real-Time PCR Master Mixes (Takara, Japan) were used for each PCR in a 20 μl reaction volume containing 1 μl of each primer (5 mM) and 4 μl of first strand cDNA. The primers used for RT-qPCR are listed in Supplementary Table 2. Actin served as an internal reference (internal control). Relative expression was calculated using the comparative Ct method 2^−ΔΔCT^ and the *C*t values of different treatments were normalized to the *C*t values of MFs which were defined as the external reference (external control). The results are expressed as the mean ± SD. The qPCR data of the blank control, negative control and RNAi treatment were analyzed by one-way ANOVA followed by Tukey’s test.

### Functional Annotation Enrichment Analysis

According to the results of the behavioral investigation, Venn diagrams of differentially expressed olfaction genes in group1 (VM/VF, MM/VF and MF/VF) and group2 (VF/VM, MF/VM and MM/VM) were constructed using Venny2.1^5^. The mean RPKM values for each gene in the different treatments (VF, MF, VM and MM) were then log-transformed using “log_2_ (RPKM + 1)” and subjected to hierarchical clustering using the minimum spanning tree; a heatmap was generated using Heml1.0 (Deng et al., 2014).

## Results

### Olfactometer Bioassay

We separately compared the taxis of VF, MF, VM, and MM to wasps of the opposite sex (10 wasps) and to the aphid alarm pheromone (2000 ng of EBF) with the taxis to the blank control. Three hundred wasps were tested for each treatment (Table 1).

**FIGURE 1:**
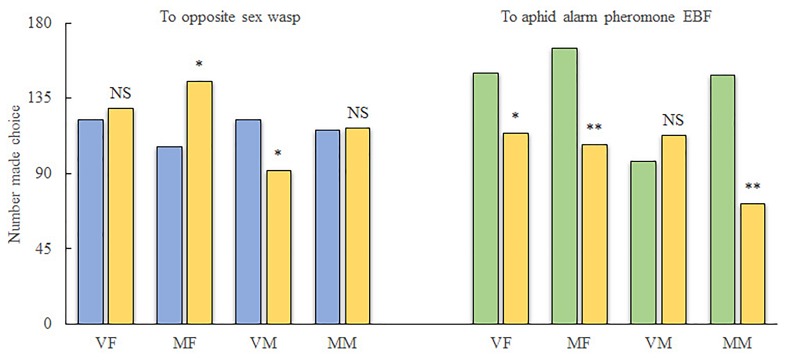
Behavioral investigation of the response of *Aphidius gifuensis* to EBF as well as to wasps of the opposite sex. VF, virgin female; MF, mated female; VM, virgin male; MM, mated male; ^∗^*P* < 0.05; ^∗∗^*P* < 0.01; NS, not significant. A total of 300 wasps were tested for each treatment. Yellow, number of wasps that chose the empty control arm; Blue, number of wasps that chose the treatment arm containing wasps of the opposite sex; Green, number of wasps that chose the treatment arm containing EBF.

For reciprocal attraction assay between sexes (Figure 1), VMs were significantly attracted to the treatment arm (10 females, χ^2^ = 4.206; df = 1; *P* < 0.05) when compared with the control arm (pure air). However, more MFs chose the control arm over the treatment arm (10 males, χ^2^ = 6.061; df = 1; *P* < 0.05). Meanwhile, MMs were indifferent to both arms (χ^2^ = 0.004; df = 1; *P* > 0.05), and VFs did not show any attraction to male wasps (χ^2^ = 0.195; df = 1; *P* > 0.05).

For the alarm pheromone (Figure 1), both VFs (χ^2^ = 4.909; df = 1; *P* < 0.05) and MFs (χ^2^ = 12.368; df = 1; *P* < 0.01) as well as MMs (χ^2^ = 26.828; df = 1; *P* < 0.01) exhibited preferences for the air from the treatment arm (2000 ng of EBF). Meanwhile, VMs exhibited weaker responses to EBF than to the control; however, the difference was not statistically significant (χ^2^ = 1.219; df = 1; *P* > 0.05).

### Combined Sequencing Approach for *A. gifuensis*

To identify and differentiate the transcriptomes of virgin and mated *A. gifuensis* of both sexes, two sequencing strategies were undertaken, using both NGS and SMRT sequencing platforms (Illumina and PacBio, respectively). First, 12 mRNA samples from four different treatments (VMs, MMs, VFs, and MFs that had emerged within the previous 24–36h; each in triplicate) were subjected to 2 × 125 paired-end sequencing using the HiSeq 2500 platform, yielding 649,863,050 reads. A total of 174178 unigenes were obtained from Illumina sequencing. Second, full-length cDNAs from 12 pooled poly(A) RNA samples were normalized and subjected to SMRT sequencing using the PacBio RS platform, Yielding a total of 518,955 raw reads. After filtering using RS_Subreads.1 of PacBio RS, 216,385 subreads were obtained. Finally, to resolve the high error rates, all subreads were corrected using the approximately 650 million NGS reads as input data. After removal of the redundant sequences for all the SMRT subreads using CD-HIT-EST (*c* = 0.85), 81,636 nonredundant reads were produced, with a mean read length of 1970 bases. Of the unigenes from NGS, 56.8% were between 200–500 bp in length, and 21.47% were more than 1 kb. However, the percentage of transcripts from SMRT between 200 and 500 bp in length was only 0.11 and that of transcripts that were more than 1 kb in length was 79.17 (Table 2).

**Table 2:** Comparison between SMRT sequencing transcripts and Illumina sequencing unigenes.

Length distribution (bp)	Illumina sequencing (unigenes)		SMRT sequencing (transcripts)	
	Number	Percentage (%)	Number	Percentage (%)
200–500	99011	56.84	93	0.11
500–1000	37761	21.68	16913	20.72
1000–2000	22837	13.11	33423	40.94
>2000	14569	8.36	31207	38.23
Total	174178	100.00	81636	100.00

### Annotation of Olfaction-Related Genes in *A. gifuensis*

**FIGURE 2:**
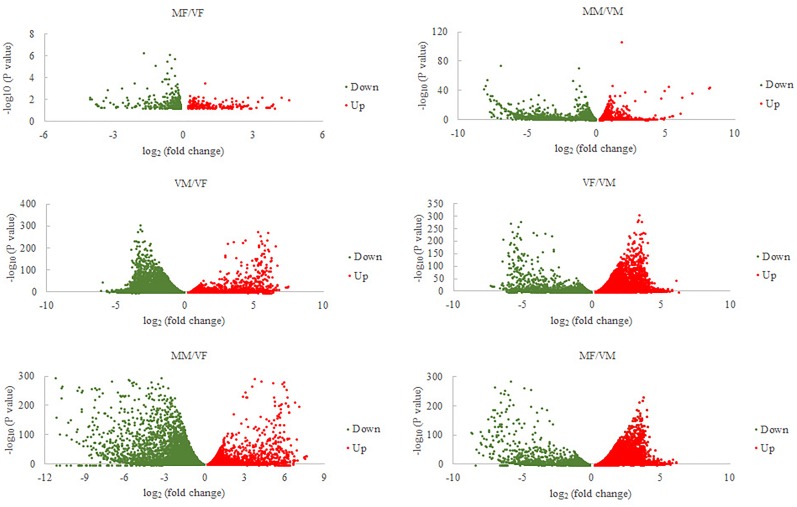
Volcano plots for differentially expressed genes between each treatment and VF or VM. VF, virgin female; MF, mated female; VM, virgin male; MM, mated male.

Sixty-six ORs, 25 IRs, 16 OBPs, and 12 CSPs were identified (GenBank accession numbers are MK048947- MK049012, MK049025- MK049049, MK049050- MK049065, MK049013- MK049024, respectively) using the NCBI BLASTX program. Gene expression analysis showed that, compared with VMs, MMs had 4060 genes that were significantly differentially expressed (MM/VM, 2515 upregulated and 1545 downregulated). The value for *A. gifuensis* females (MF/VF) was 556 (219 upregulated and 337 downregulated), the VF/VM value was 12608 (7253 upregulated and 5355 downregulated), MF/VM was 19185 (7000 upregulated and 12185 downregulated), VM/VF was 12608 (5355 upregulated and 7253 downregulated), and MM/VF was 13151 (5669 upregulated and 7482 downregulated) (Figure 2).

### Differential Expression Analysis of Olfaction Genes

**FIGURE 3:**
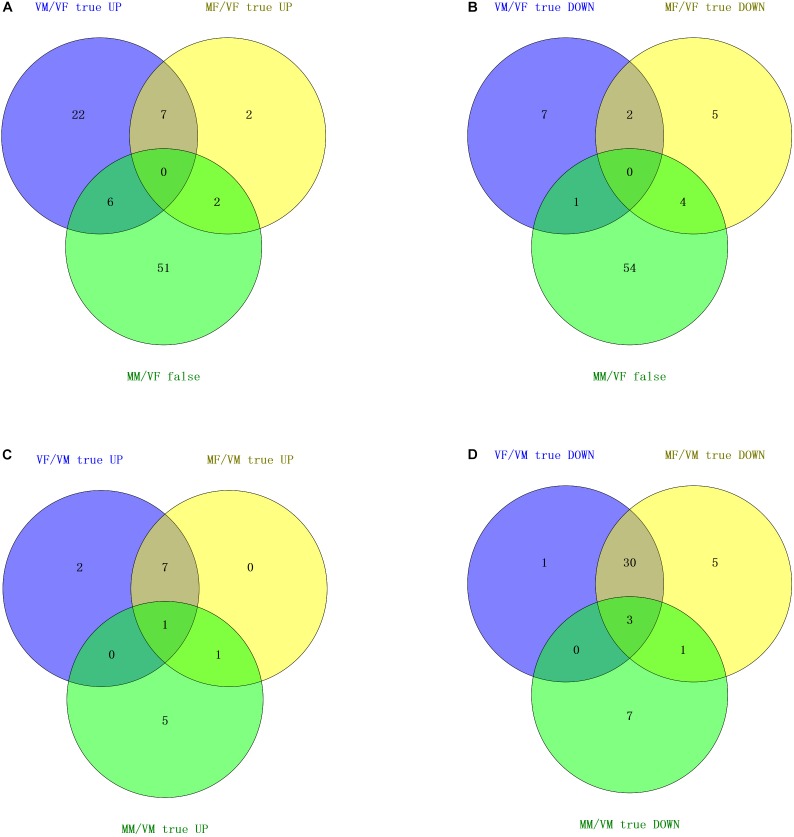
Venn diagram based on a combined analysis of differential expression of transcripts and behavioral investigations. **(A,B)** According to the behavioral test results for the response to wasps of the opposite sex, the intersection of differentially expressed olfactory genes in VM/VF and comparable olfactory genes in MM/VF contains five genes (upregulated) and one gene (downregulated), which represent six candidate genes that may be involved in the recognition of the opposite sex by VMs. The intersection of differentially expressed olfactory genes in MF/VF and comparable olfactory genes in MM/VF contains three genes (upregulated) and four genes (downregulated), respectively, which represents seven candidate genes that could be involved in the recognition of the opposite sex by MFs. **(C,D)** According to the results of the behavioral test for the response to EBF, the number of common up- and downregulated olfactory genes was 1 and 3, respectively, which represent four candidate genes that may be involved in EBF perception.

Based on the behavioral test results (see details in the behavioral investigation section), differentially expressed olfaction genes between treatments were compared with VFs or VMs (Supplementary Table 1) and analyzed using Venn diagrams (Figure 3).

Neither VFs nor MMs exhibited a behavioral response to *A. gifuensis* wasps of the opposite sex. However, VMs were attracted by females, and MFs were repelled by males. We firstly chose common olfactory genes with comparable expression levels (no statistically significant differences) between VFs and MMs and denoted these genes as “MM/VF false” (*P* > 0.05). Then, the differentially expressed olfactory genes (both up- and downregulated) in MF/VF as well as VM/VF were separately compared with “MM/VF false.” The final Venn diagram showed seven common genes between “VM/VF true” and “MM/VF false,” six of which (4 ORs: c55179_g2, c53716_g5, c53086_g3, c34269_g1; 1 IR: c46617_g3, and 1 OBP: c55239_g5) were present exclusively in two gene sets, namely, “VM/VF true UP” and “MM/VF false” (Figure 3A), and the other gene (Figure 3B, 1 IR: c56684_g4) was present exclusively in “VM/VF true DOWN” and “MM/VF false”; “MF/VF true” and “MM/VF false” shared six common genes, two of which (2 ORs: c53272_g1 and c51725_g3) were present exclusively in “MF/VF true UP” and “MM/VF false,” and four of which (2 ORs: c58301_g1 and c57979_g1; 1 IR: c50331_g1; and 1 CSP: c55251_g3) were present exclusively in sets “MF/VF true DOWN” and “MM/VF false.”

MFs, MMs, and VFs were strongly attracted by EBF. However, VMs were indifferent to EBF. Therefore, we selected olfaction genes that were differentially expressed in MFs, MMs, and VFs compared separately with the expression levels in VM (“MF/VM true UP/DOWN,” “MM/VM true UP/DOWN,” and “VF/VM true UP/DOWN,” *P* < 0.05; Figures 3C,D). The intersection of the Venn diagram showed 1 common gene in the 3 “UP” gene sets (“MF/VM true UP,” “MM/VM true UP,” and “VF/VM true UP”), namely, c56684_g4 (IR), and three common genes in the 3 “DOWN” gene sets (“MF/VM true DOWN,” “MM/VM true DOWN,” and “VF/VM true DOWN”), namely c34269_g1, c46617_g3 and c55239_g5 (1 OR, 1 IR, and 1 OBP, respectively). In summary, the following observations were made based on our behavioral test results: (I) Both MFs and VMs exhibited behavioral responses to wasps of the opposite sex but the responses were opposite. *A. gifuensis* MF exhibited a lower preference for *A. gifuensis* males than for the control arms, indicating that MFs are repelled by the males. VMs were attracted by females. Meanwhile, MMs and VFs showed no preference for *A. gifuensis* wasps of the opposite sex (χ^2^ test, *P* > 0.05, Figure 1). The common olfactory genes expressed in both MMs and VFs at comparable levels (*P* > 0.05) were pooled as the “MM/VF false” set. The sets of differentially expressed genes in VMs and MFs compared with VFs were screened and named “VM/VF true” and “MF/VF true,” respectively. Seven common genes in “VM/VF true” and “MM/VF false,” but not in “MF/VF true,” were found to be candidate genes involved in the positive behavioral response of VMs to female wasps (4 ORs: c55179_g2, c53716_g5, c53086_g3, c34269_g1; 2 IR: c46617_g3, c56684_g4; and 1 OBP: c55239_g5), and six common genes in “MF/VF true” and “MM/VF false,” but not in “VM/VF true,” were found to be candidate genes involved in the negative behavioral response of MFs to male wasps (4 ORs: c53272_g1, c51725_g3, c58301_g1 and c57979_g1; 1 IR: c50331_g1; and 1 CSP: c55251_g3). (II) MFs, VFs, and MMs exhibited chemotaxis toward the aphid alarm pheromone EBF, whereas VMs did not respond. Genes that were differentially expressed in MFs, VFs, and MMs when compared with VMs are shown in a Venn diagram. Four olfaction genes were screened as candidate genes involved in EBF perception (1 OR: c34269_g1; 2 IRs: c46617_g3, c56684_g4, and 1 OBP: c55239_g5). (III) Four genes, namely, c34269_g1, c46617_g3, c56684_g4 and c55239_g5 (1 OR, 2 IRs and 1 OBP,), were screened out simultaneously based on the two strategies above; these genes are candidate genes involved in the perception of both the aphid alarm pheromone and *A. gifuensis* sex pheromone.

The results of differential expression analysis were then verified by qPCR (two randomly selected olfactory genes form each family, see details in Supplementary Figure 1). For example, qPCR data showed that CSP c55251 was more highly expressed in both VFs and VMs than MFs and MMs. With no significant difference between VFs and VMs. These results are consistent with the above results from the differential expression analysis of olfaction genes based on the transcriptome sequencing data.

### Functional Annotation Enrichment Analysis

**FIGURE 4:**
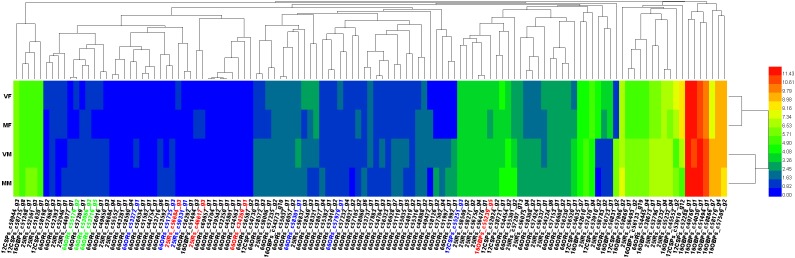
Heatmap of all the annotated olfactory genes. Genes marked in green, three of six candidate genes involved in recognition of the opposite sex by VMs; blue, six candidate genes involved in recognition of the opposite sex by MFs; red, the remaining four of seven candidate genes involved in recognition of the opposite sex by VMs, which are also involved in EBF perception.

The gene dendrograms showed many clusters of olfactory genes (Figure 4), which was consistent with the various functions of these genes in the detection and transmission of olfactory signals from the environment.

The heat map (Figure 4) showed that all 4 candidate genes for EBF perception (in VFs, MFs, and MMs, c34269_g1, c46617_g3, c56684_g4 and c55239_g5) were shared with female volatile perception, which implied that these four genes could participate in either or both physiological processes. The remaining three of the seven candidate genes for female volatile perception (in MMs) were mainly clustered together. In contrast, the six candidate genes for male smell perception (in MFs) were distributed widely across the heatmap. Compared with ORs, OBPs, and CSPs did not show much clustering, which may indicate their generalist nature in ligand binding.

## Discussion

In this study, we carried out, for the first time, two olfactory behavioral investigations, one examining the response to wasps of the opposite sex and the other examining the response to EBF, the alarm pheromone from aphids. Furthermore, wasps were distinguishing by mating status (virgin or mated) rather than simply according sex (male and female) to reveal additional details of *A. gifuensis* mating and predatory behaviors.

For the reciprocal attraction assay between sexes, VMs were attracted by females, which demonstrated the secretion of a volatile sex pheromone by females. However, MMs did not respond to female volatiles. In addition to olfaction, vision is also believed to be very important to insects (Reeves, 2011). Additionally, the learning ability of *Aphidius* has been widely reported (e.g., Takemoto et al., 2012). Therefore, multiple sensory behaviors in males, such as olfaction, gustation and vision, likely participate in the recognition of females. With increasing experience, MMs may eventually employ other modes of sensory perception, most likely vision and/or taste, rather than depending solely on olfaction.

All treatment groups except VMs were significantly attracted by EBF. As a common aphid alarm pheromone, EBF signals a high risk to aphids’ survival and generally repels aphids. EBF can be used by organisms at high trophic levels as a kairomone to detect and locate aphids (e.g., Micha and Wyss, 1996). *A. gifuensis* is known to be an egg parasitoid of aphids, and the ability to track EBF from aphids may enhance the parasite searching behavior of females. This ability can also be helpful when searching for a female mate. However, the results showed that, in contrast to MMs, VMs do not respond to EBF. This finding indicates that after their first mating activity, *A. gifuensis* males likely exploit strategies other than simply responding to female smells (e.g., sex pheromone attraction). Considering that males do not prey on aphids and that the main role of the males is mating, the positive taxis of MMs to EBF may be an evolutional adaptation for locating aphids, further increasing the chances of encountering a potential mate. However, the exact reason that males stop responding to female smells after the first mating remains unclear.

This is the first study to document a repellent response of mated *A. gifuensis* females to males. The mechanism may be quite complicated. Females mate only once during their life cycle, whereas males continuously attempt courtship and to mate with any female, even those that have already mated. Therefore, vigilance against males from a distance is more effective than detecting males upon touch.

We also described, for the first time, four cDNA libraries from VM, MM, VF, and MF *A. gifuensis* wasps using transcriptomes obtained via a combination of NGS and SMRT sequencing. A total of 66 ORs, 25 IRs, 16 OBPs, and 12 CSPs were annotated, and some genes with potentially important functions were further pooled based on the olfactory behavioral investigations mentioned above.

The superiority of SMRT sequencing, which can produce full-length transcripts, compared with short-read sequencing methods has been demonstrated in various species, including humans (Sharon et al., 2013). In the present study, most transcripts (79.17%) were longer than 1 kb, and only 0.11% of the transcripts were between 0–500 bp in length. In contrast, most unigenes (78.52%) from NGS were shorter than 1 kb, and the percentage of unigenes between 0–500 bp was up to 56.84%.

A total of six candidate genes (Figure 4, genes are shown in both green and red) were found to be involved in the perception of wasps of the opposite sex by MFs, including four ORs, which were distributed separately on the heatmap. This result suggested that multiple infochemicals help mated females to avoid physical contact with males.

Notably, all four candidate genes associated with EBF perception were present in VMs for female smell perception. This finding may imply a closer evolutionary relationship between genes for perceiving pheromones than between those for normal odor detection.

Substantial progress has been made in studies of insect olfaction mechanisms since ApolOBP, the first functional insect olfactory protein, was identified in *Antheraea polyphemus* (Vogt and Riddiford, 1981). However, the functional analysis based on 2nd + 3rd generation sequencing and behavioral investigation reported here, particularly the behavioral investigation of VF, MF, VM, MM *A. gifuensis* wasps, is novel. Our results identified differences in both olfactory responses to certain volatiles and the expression of the corresponding olfactory genes between “before” and “after” mating in males or females; thirteen candidate genes that are potentially involve in EBF and sex pheromone perception were identified from 119 olfactory genes (66 ORs, 25 IRs, 16 OBPs, and 12 CSPs). This approach provides reliable transcript information including coding sequences and expression levels, which have been verified by gene cloning (Au et al., 2013) and qPCR (in the present paper), respectively. Our study definitively provides valuable information for understanding olfaction in *A. gifuensis* at the molecular level, which will help to strengthen and even take better advantage of *A. gifuensis* as a powerful and natural biocontrol strategy.

## Author Contributions

JF conceived and designed the study, helped to perform the experiments, analyzed the data, and wrote the paper. QZ helped with RNA extraction as well as data analysis, and revised the manuscript. QX, WX, and ZH performed the behavioral tests and statistical analysis. JS discussed the data and revised the manuscript. JC organized and directed the project.

## Conflict of Interest Statement

The authors declare that the research was conducted in the absence of any commercial or financial relationships that could be construed as a potential conflict of interest.

## Footnotes

1 http://www.pacificbiosciences.com

2 http://www.clontech.com

3 http://www.evrogen.com

4 http://www.beckmancoulter.com

5 http://bioinfogp.cnb.csic.es/tools/venny/index.html

## References

[B1] Au K. F. Sebastiano V. Afshar P. T. (2013). Characterization of the human ESC transcriptome by hybrid sequencing. *Proc. Natl. Acad. Sci. U.S.A.* 110 4821–4830. 10.1073/pnas.1320101110 24282307 PMC3864310

[B2] Benton R. Sachse S. Michnick S. W. Vosshall L. B. (2006). Atypical membrane topology and heteromeric function of Drosophila odorant receptors *in vivo*. *PLoS Biol.* 4:e20. 10.1371/journal.pbio.0040020 16402857 PMC1334387

[B3] Benton R. Vannice K. S. Gomez-Diaz C. Vosshall L. B. (2009). Variant ionotropic glutamate receptors as chemosensory receptors in Drosophila. *Cell* 136 149–162. 10.1016/j.cell.2008.12.001 19135896 PMC2709536

[B4] Bi Z. Ji Z. (1994). Bionomics of *Aphidius gifuensis* Ashmead II. Bionomics of adult and over winter. *J. Hebei Agric. Univ.* 17 38–44.

[B5] Clyne P. J. Warr C. G. Freeman M. R. Lessing D. Kim J. H. Carlson J. R. (1999). A novel family of divergent seven-transmembrane proteins: candidate odorant receptors in Drosophila. *Neuron* 22 327–338. 10.1016/S0896-6273(00)81093-4 10069338

[B6] Deng W. Wang Y. Liu Z. Cheng H. Xue Y. (2014). HemI: a toolkit for illustrating heatmaps. *PLoS One* 9:e111988. 10.1371/journal.pone.0111988 25372567 PMC4221433

[B7] Fan J. Zhang Y. Francis F. Cheng D. Sun J. Chen J. (2015). Orco mediates olfactory behaviors and winged morph differentiation induced by alarm pheromone in the grain aphid, sitobion avenae. *Insect Biochem. Mol.* 64 16–24. 10.1016/j.ibmb.2015.07.006 26187252

[B8] Hackl T. Hedrich R. Schultz J. Forster F. (2014). proovread: large-scale high-accuracy PacBio correction through iterative short-read consensus. *Bioinformatics* 30 3004–3011. 10.1093/bioinformatics/btu392 25015988 PMC4609002

[B9] Kang Z. Tian H. Liu F. Liu X. Jing X. Liu T. (2017). Identification and expression analysis of chemosensory receptor genes in an aphid endoparasitoid *Aphidius gifuensis*. *Sci. Rep.* 7:3939. 10.1038/s41598-017-03988-z 28638084 PMC5479799

[B10] Li B. Dewey C. N. (2011). RSEM: accurate transcript quantification from RNA-Seq data with or without a reference genome. *BMC Bioinformatics* 12:323. 10.1186/1471-2105-12-323 21816040 PMC3163565

[B11] Micha S. G. Wyss U. (1996). Aphid alarm pheromone (E)-β-farnesene: a host finding kairomone for the aphid primary parasitoid *Aphidius uzbekistanicus* (Hymenoptera: Aphidiinae). *Chemoecology* 7 132–139. 10.1007/BF01245965

[B12] Mondor E. B. Baird D. S. Slessor K. N. Roitberg B. D. (2000). Ontogeny of alarm pheromone secretion in pea aphid, Acyrthosiphon pisum. *J. Chem. Ecol.* 26 2875–2882. 10.1023/A:1026402229440

[B13] Ohta I. Honda K. (2010). Use of Sitobion akebiae (hemiptera: aphididae) as an alternative host aphid for a banker-plant system using an indigenous parasitoid, *Aphidius gifuensis* (hymenoptera: braconidae). *Appl. Entomol. Zool.* 45 233–238. 10.1303/aez.2010.233

[B14] Pelosi P. Zhou J. Ban L. Calvello M. (2006). Soluble proteins in insect chemical communication. *Cell Mol. Life Sci.* 63 1658–1676. 10.1007/s00018-005-5607-0 16786224 PMC11136032

[B15] Reeves J. R. (2011). Vision should not be overlooked as an important sensory modality for finding host plants. *Environ. Entomol.* 40 855–863. 10.1603/EN10212 22251686

[B16] Sharon D. Tilgner H. Grubert F. Snyder M. (2013). A single-molecule long-read survey of the human transcriptome. *Nat. Biotechnol.* 31 1009–1014. 10.1038/nbt.2705 24108091 PMC4075632

[B17] Takemoto H. Powell F. Pickett J. Kainoh Y. Takabayashi J. (2012). Two-step learning involved in acquiring olfactory preferences for plant volatiles by parasitic wasps. *Anim. Behav.* 83 1491–1496. 10.1016/j.anbehav.2012.03.023

[B18] Tan X. L. Liu T. X. (2014). Aphid-induced plant volatiles affect the attractiveness of tomato plants to *Bemisia tabaci* and associated natural enemies. *Entomol. Exp. App.* 151 259–269. 10.1111/eea.12190

[B19] Trapnell C. Roberts A. Goff L. Pertea G. Kim D. Kelley D. R. (2012). Differential gene and transcript expression analysis of RNA-seq experiments with TopHat and Cuffiinks. *Nat. Protoc.* 7 562–578. 10.1038/nprot.2012.016 22383036 PMC3334321

[B20] Vogt R. G. Riddiford L. M. (1981). Pheromone binding and inactivation by moth antennae. *Nature* 293 161–163. 10.1038/293161a0 18074618

