# Supplementary material for: Differential Expression Analysis of Olfactory Genes Based on a Combination of Sequencing Platforms and Behavioral Investigations in Aphidius gifuensis
Source: Front Physiol. 2018 Nov 27;9:1679. doi: 10.3389/fphys.2018.01679 (PMC6277867; doi:10.3389/fphys.2018.01679)
Supplement: Supplementary file 1 [file Image_1.pdf]

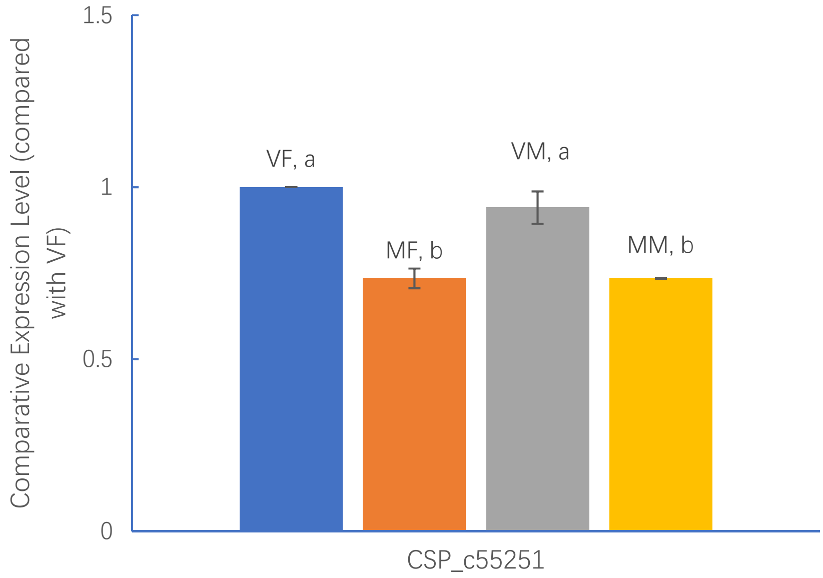
1.5
1
0.5
0
Comparative Expression Level (compared with VF)
VF, a
MF, b
VM, a
MM, b
CSP_c55251

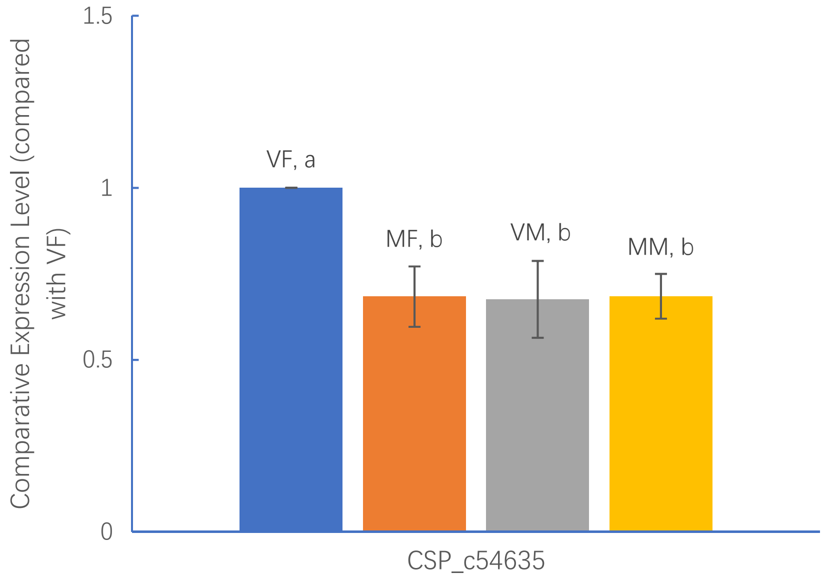
1.5
1
0.5
0
Comparative Expression Level (compared with VF)
VF, a
MF, b
VM, b
MM, b
CSP_c54635

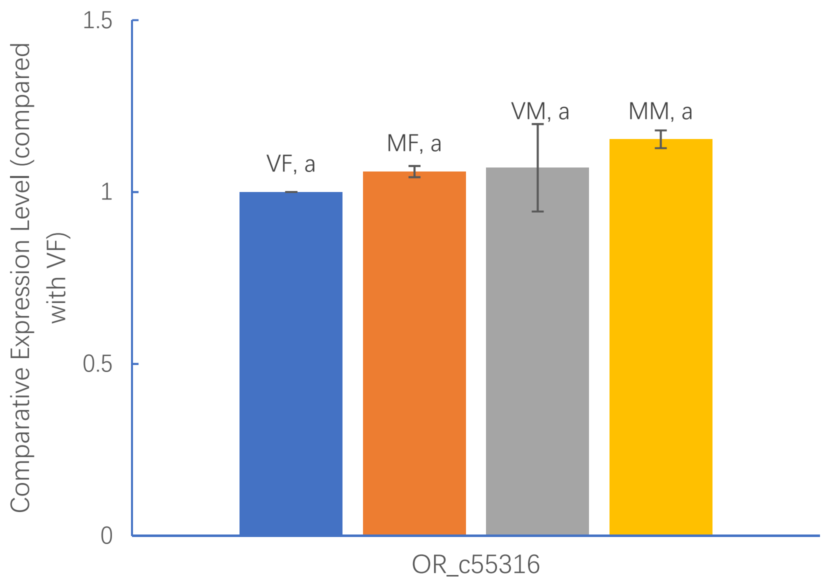
1.5
1
0.5
0
Comparative Expression Level (compared with VF)
VF, a
MF, a
VM, a
MM, a
OR_c55316

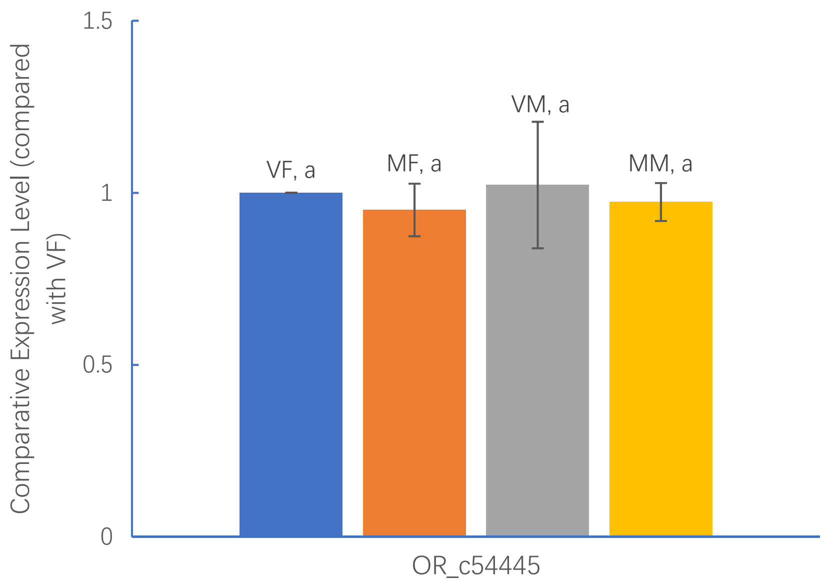
1.5
1
0.5
0
Comparative Expression Level (compared with VF)
VF, a
MF, a
VM, a
MM, a
OR_c54445

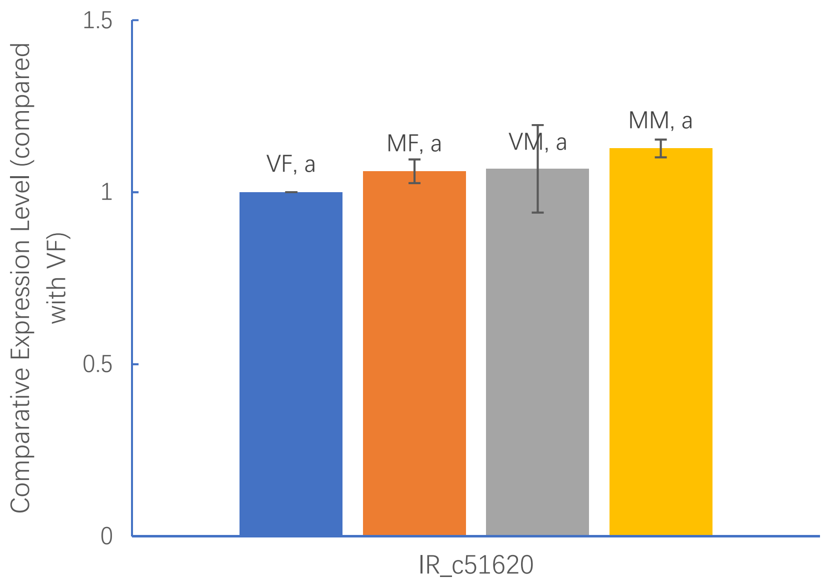

1.5
1
0.5
0
Comparative Expression Level (compared with VF)
VF, a
MF, a
VM, a
MM, a
IR_c51620

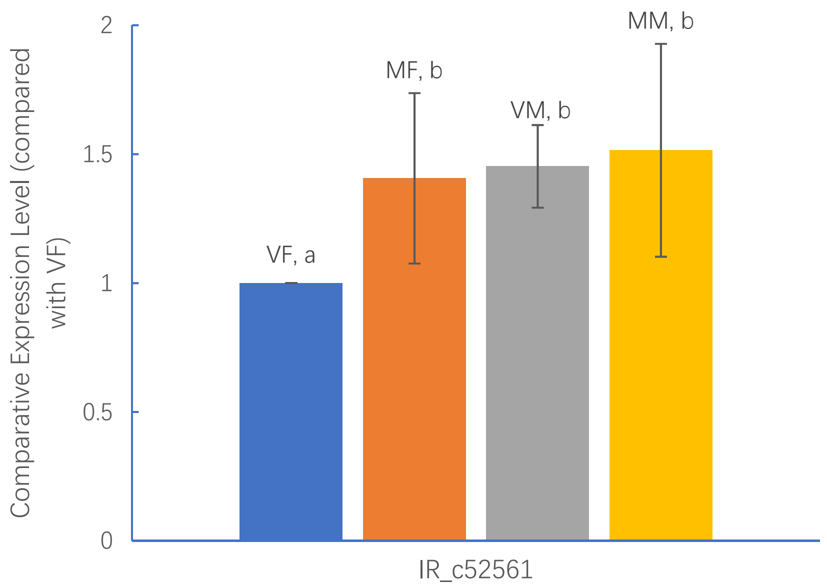

2
1.5
1
0.5
0
Comparative Expression Level (compared with VF)
VF, a
MF, b
VM, b
MM, b
IR_c52561

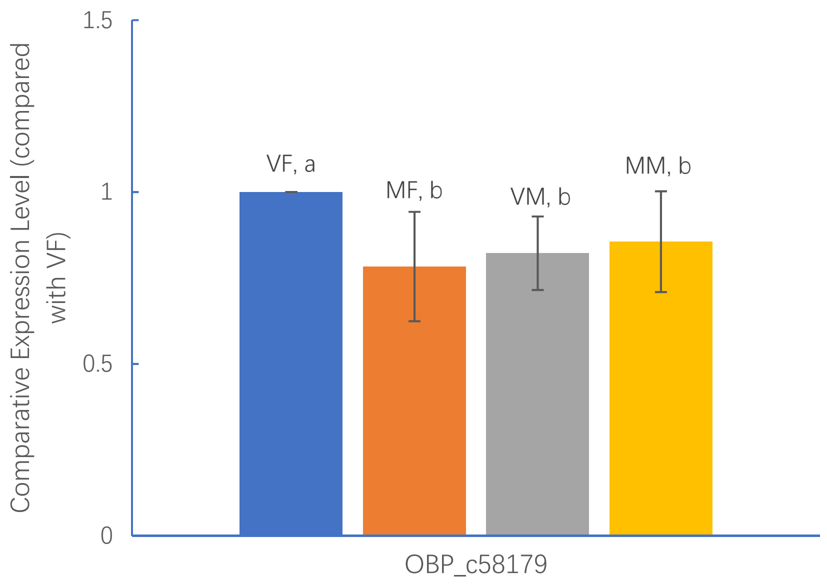

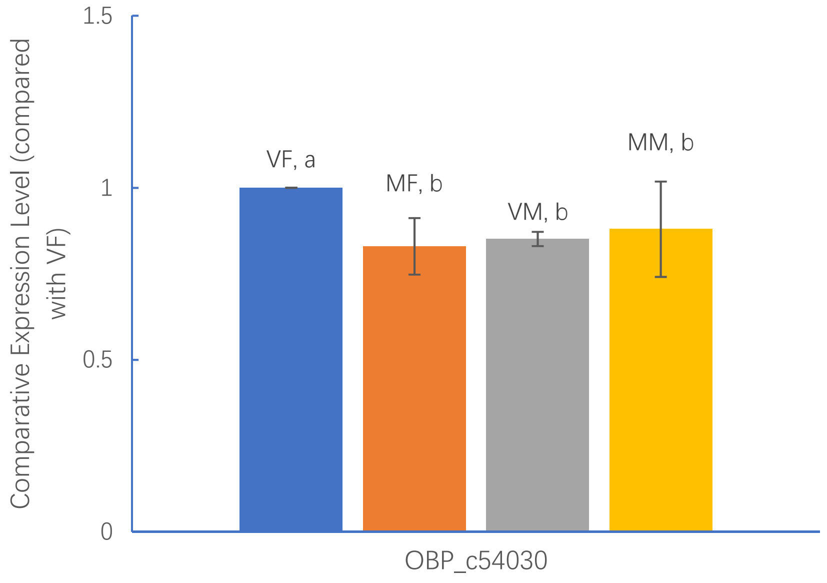

Supplementary figure 1 qPCR verification on 8 randomly selected olfactory genes
Data were compared and tested by ANOVA followed by Duncan's new multiple range test at $p < 0.05$ level
