# Supplementary material for: Differential Expression Analysis of Olfactory Genes Based on a Combination of Sequencing Platforms and Behavioral Investigations in Aphidius gifuensis
Source: Front Physiol. 2018 Nov 27;9:1679. doi: 10.3389/fphys.2018.01679 (PMC6277867; doi:10.3389/fphys.2018.01679)
Supplement: Supplementary file 2 [file Table_1.docx]

Supplementary Table 1 differential expressed olfactory genes list. VF: virgin female. MF: mated female. VM: virgin male. MM: mated male. VIRGIN: both virgin female and virgin male. MATED: both mated female and mated male. SE: specific expression. AE: absent expression.

| Compare with VM | | | | |
| --- | --- | --- | --- | --- |
|  | up (P<0.05) | down (P<0.05) | specific expression（SE） | illustration |
| MM | 66 ORs c34563_g1  25 IRs c56684_g4  25 IRs c52561_g3  16 OBPs c54030_g1  16 OBPs c51494_g3  16 OBPs c51398_g1  12 CSPs c57906_g2 | 66 ORs c58677_g4  66 ORs c56723_g1  66 ORs c56587_g2  66 ORs c56292_g1  66 ORs c49472_g1  66 ORs c34269_g1  25 IRs c56684_g3  25 IRs c50331_g1  16 OBPs c57509_g2  16 OBPs c55239_g5 | 66 ORs c34563_g1  66 ORs c56292_g1  66 ORs c56587_g2  25 IRs c56684_g3  16 OBPs c51494_g3 | MM SE  MM AE  VIRGIN SE  MM AE  MATED SE |
| VF | 66 ORs c58677_g4  66 ORs c56723_g1  66 ORs c56230_g1  25 IRs c56684_g4  25 IRs c58783_g2  25 IRs c57470_g4  25 IRs c56337_g1  16 OBPs c49769_g1  16 OBPs c45552_g2  12 CSPs c53910_g2  12 CSPs c53910_g12 | 66 ORs c58590_g4  66 ORs c58572_g3  66 ORs c58126_g2  66 ORs c57502_g4  66 ORs c57424_g1  66 ORs c56628_g7  66 ORs c56520_g1  66 ORs c55301_g2  66 ORs c55179_g2  66 ORs c55035_g5  66 ORs c54819_g2  66 ORs c54454_g2  66 ORs c54445_g4  66 ORs c53716_g6  66 ORs c53716_g5  66 ORs c53592_g1  66 ORs c53086_g3  66 ORs c51997_g1  66 ORs c51992_g1  66 ORs c49472_g1  66 ORs c45801_g1  66 ORs c43281_g1  66 ORs c34269_g1  25 IRs c58232_g4  25 IRs c56493_g2  25 IRs c53796_g1  16 OBPs c58869_g1  16 OBPs c58866_g7  16 OBPs c56831_g2  16 OBPs c55239_g5  16 OBPs c54030_g1  16 OBPs c51494_g1  16 OBPs c38673_g1  12 CSPs c54730_g3 | 66 ORs c58677_g4  66 ORs c49472_g1 | VF AE  VF AE |
| MF | 66 ORs c56723_g1  25 IRs c56684_g4  25 IRs c58783_g2  25 IRs c57470_g4  25 IRs c46617_g3  16 OBPs c51494_g3  16 OBPs c49769_g1  16 OBPs c45552_g2  12 CSPs c53910_g2  12 CSPs c53910_g12 | 66 ORs c58590_g4  66 ORs c58572_g3  66 ORs c58126_g2  66 ORs c57502_g4  66 ORs c57424_g1  66 ORs c56628_g7  66 ORs c56587_g2  66 ORs c56520_g1  66 ORs c55301_g2  66 ORs c55179_g2  66 ORs c55035_g5  66 ORs c54819_g2  66 ORs c54454_g2  66 ORs c54445_g4  66 ORs c53716_g6  66 ORs c53716_g5  66 ORs c53592_g1  66 ORs c53272_g1  66 ORs c51997_g1  66 ORs c51992_g1  66 ORs c51725_g3  66 ORs c51119_g1  66 ORs c45801_g1  66 ORs c43281_g1  66 ORs c34269_g1  25 IRs c58232_g4  25 IRs c56837_g3  25 IRs c56493_g2  25 IRs c53796_g1  16 OBPs c58869_g1  16 OBPs c58866_g7  16 OBPs c56831_g2  16 OBPs c55239_g5  16 OBPs c54030_g1  16 OBPs c51494_g1  16 OBPs c38673_g1  12 CSPs c55251_g3  12 CSPs c54730_g3 | 66 ORs c53272_g1  66 ORs c51992_g1  66 ORs c51725_g3  25 IRs c46617_g3  16 OBPs c51494_g3 | MF AE  MF AE  MF AE  MF SE  MATED SE |
| Compare with VF | | | | |
|  | up (P<0.05) | down (P<0.05) | specific expression（SE） | illustration |
| MF | 66 ORs c58590_g4  66 ORs c57502_g4  66 ORs c53592_g1  66 ORs c53272_g1  66 ORs c51997_g1  66 ORs c51992_g1  66 ORs c51725_g3  66 ORs c45801_g1  66 ORs c43281_g1  25 IRs c46617_g3  16 OBPs c56831_g2  16 OBPs c51494_g3 | 66 ORs c58677_g4  66 ORs c58301_g1  66 ORs c57979_g1  66 ORs c56587_g2  66 ORs c51992_g1  66 ORs c49472_g1  25 IRs c56837_g3  25 IRs c56493_g2  25 IRs c56337_g1  16 OBPs c45552_g2  12 CSPs c55251_g3 | 66 ORs c53272_g1  66 ORs c51992_g1  66 ORs c51725_g3  25 IRs c46617_g3  16 OBPs c51494_g3 | MF AE  MF AE  MF AE  MF SE  MATED SE |
| VM | 66 ORs c58677_g4  66 ORs c58590_g4  66 ORs c58572_g3  66 ORs c58126_g2  66 ORs c57502_g4  66 ORs c57424_g1  66 ORs c56628_g7  66 ORs c56520_g1  66 ORs c55301_g2  66 ORs c55179_g2  66 ORs c55035_g5  66 ORs c54819_g2  66 ORs c54454_g2  66 ORs c54445_g4  66 ORs c53716_g6  66 ORs c53716_g5  66 ORs c53592_g1  66 ORs c53086_g3  66 ORs c51997_g1  66 ORs c49472_g1  66 ORs c45801_g1  66 ORs c43281_g1  66 ORs c34269_g1  25 IRs c58232_g4  25 IRs c56493_g2  25 IRs c53796_g1  16 OBPs c58869_g1  16 OBPs c58866_g7  16 OBPs c56831_g2  16 OBPs c55239_g5  16 OBPs c54030_g1  16 OBPs c51494_g1  16 OBPs c38673_g1  12 CSPs c54730_g3 | 66 ORs c56723_g1  66 ORs c56230_g1  25 IRs c56684_g4  25 IRs c58783_g2  25 IRs c57470_g4  25 IRs c56337_g1  16 OBPs c49769_g1  16 OBPs c45552_g2  12 CSPs c53910_g2  12 CSPs c53910_g12 | 66 ORs c57502_g4  66 ORs c53592_g1  66 ORs c51997_g1  66 ORs c45801_g1  66 ORs c43281_g1  66 ORs c34269_g1  25 IRs c56684_g4  16 OBPs c56831_g2 | MALE SE  MALE SE  MALE SE  MALE SE  MALE SE  VM SE  VM AE  MALE SE |
| MM | 66 ORs c58677_g4  66 ORs c58590_g4  66 ORs c58572_g3  66 ORs c58308_g2  66 ORs c58126_g2  66 ORs c57502_g4  66 ORs c57424_g1  66 ORs c57235_g2  66 ORs c56628_g7  66 ORs c56520_g1  66 ORs c55301_g2  66 ORs c55035_g5  66 ORs c54819_g2  66 ORs c54454_g2  66 ORs c54445_g4  66 ORs c53716_g6  66 ORs c53592_g1  66 ORs c51997_g1  66 ORs c51992_g1  66 ORs c51119_g1  66 ORs c49472_g1  66 ORs c45801_g1  66 ORs c43281_g1  66 ORs c34563_g1  25 IRs c58509_g2  25 IRs c58232_g4  25 IRs c56493_g2  25 IRs c55232_g4  25 IRs c53796_g1  25 IRs c52561_g3  16 OBPs c58869_g1  16 OBPs c58866_g7  16 OBPs c56831_g2  16 OBPs c56133_g19  16 OBPs c54030_g1  16 OBPs c51494_g3  16 OBPs c51494_g1  16 OBPs c51398_g1  16 OBPs c42610_g1  16 OBPs c38673_g1  12 CSPs c57906_g2  12 CSPs c54730_g3  12 CSPs c52288_g1 | 66 ORs c56723_g1  66 ORs c56587_g2  66 ORs c56292_g1  66 ORs c56230_g1  25 IRs c56684_g3  25 IRs c50331_g1  25 IRs c58783_g2  25 IRs c58271_g2  25 IRs c57470_g4  25 IRs c56337_g1  16 OBPs c57509_g2  16 OBPs c49769_g1  16 OBPs c45552_g2  12 CSPs c53910_g2  12 CSPs c53910_g12 | 66 ORs c58590_g4  66 ORs c57502_g4  66 ORs c53592_g1  66 ORs c51997_g1  66 ORs c45801_g1  66 ORs c43281_g1  66 ORs c34563_g1  66 ORs c56292_g1  25 IRs c56684_g3  25 IRs c50331_g1  16 OBPs c56831_g2  16 OBPs c51494_g3 | MALE SE  MALE SE  MALE SE  MALE SE  MALE SE  MALE SE  MM SE  MM AE  MM AE  MM AE  MALE SE  MATED SE |
