# Supplementary material for: Differential Expression Analysis of Olfactory Genes Based on a Combination of Sequencing Platforms and Behavioral Investigations in Aphidius gifuensis
Source: Front Physiol. 2018 Nov 27;9:1679. doi: 10.3389/fphys.2018.01679 (PMC6277867; doi:10.3389/fphys.2018.01679)
Supplement: Supplementary file 3 [file Table_2.DOCX]

Supplementary table 2. Primers for qRT-PCR

| Primer | Sequence (5'- 3') |
| --- | --- |
| OBPs_c58179-F | CAGCGGAGGCAGTTGTAACAGTAG |
| OBPs_c58179-R | GATCCAGCCACTTCGCACTCG |
| OBPs_c54030-F | TGGTGCATTGACTCCTGAGC |
| OBPs_c54030-R | CCACTGTTGTCCATCAGTCCT |
| IRs_c52561-F | TGCACCAGTTGCTCAAGACA |
| IRs_c52561-R | TGCTGGTGTTGATTTTGCACC |
| IRs_c51620-F | ATGGTGTCGGTGCATGTGGTTG |
| IRs_c51620-F | ACGTGGTGTACCTTCGCCAATTC |
| CSPs_c54635-F | GCAATGACTGTACCACCGGAACC |
| CSPs_c54635-R | GTTGAGCAAGACGAGGCATTGTTG |
| CSPs_c55251-F | GTGAAGCTGAACCACGTCGAA |
| CSPs_c55251-R | ACCACCAACATCACGCCAAC |
| ORs_c55316-F | GCTGGTGTCTCAATGAGCTGGAAG |
| ORs_c55316-R | TGGCATGATGATTCGTGGTGCTAC |
| ORs_c54445-F | GCTCAGTGCAACACTTGACACAG |
| ORs_c54445-R | TGGTGGAGCAGTTCCAGTTGTATC |
| β_actin-F | CGTTACCAACTGGGACGATATG |
| β_actin-R | GGGTTCAATGGAGCTTCTGTTA |
